# The Examination of Diffusion Effects on Modern Contraceptive Use in Nigeria

**DOI:** 10.1007/s13524-020-00884-6

**Published:** 2020-05-19

**Authors:** David K. Guilkey, Veronica Escamilla, Lisa M. Calhoun, Ilene S. Speizer

**Affiliations:** 1 Carolina Population Center, University of North Carolina at Chapel Hill, Chapel Hill, NC 27599-3305 USA; 2 Department of Economics, University of North Carolina at Chapel Hill, Chapel Hill, NC 27599-3305 USA; 3 Department of Maternal and Child Health, University of North Carolina at Chapel Hill, Chapel Hill, NC 27599-3305 USA

**Keywords:** Diffusion, Modern contraceptive use, Nigeria, Dynamic model estimation

## Abstract

This study uses data gathered for an evaluation of a Bill & Melinda Gates Foundation–funded initiative designed to increase modern contraceptive use in select urban areas of Nigeria. When the initiative was conceived, the hope was that any positive momentum in the cities would diffuse to surrounding areas. Using a variety of statistical methods, we study three aspects of diffusion and their effects on modern contraceptive use: spread through mass communications, social learning, and social influence. Using a dynamic causal model, we find strong evidence of social multiplier effects through social learning. The results for social influence and spread through mass communications are promising, but we are unable to identify definitive causal impacts.

**Electronic supplementary material:**

The online version of this article (10.1007/s13524-020-00884-6) contains supplementary material, which is available to authorized users.

## Introduction

Access to modern contraceptive services and promotion of these services are important components of programs aimed at improving maternal and child health outcomes, and can facilitate meeting the Sustainable Development Goals (SDG) of the United Nations (Cleland et al. 2012; Starbird et al. 2016; United Nations Population Fund (UNPFA) 2016). Evaluations of these programs typically involve measures of program effectiveness based on assessing an individual’s direct exposure to program activities. These evaluations may understate true program impact if program components have indirect effects through other pathways. For example, information about the program can spread by people talking to one another about what they learned at a clinic; by mobility of people from outside target sites into program areas; or through T-shirts with logos spreading to outside intervention sites, among other things. There also may be indirect or multiplier effects through the pathways of social interactions and changing social norms (see, e.g., Montgomery and Casterline 1996).

These indirect pathways are rarely measured in family planning (FP) program evaluations for several reasons. First, data are rarely collected outside the target area to assess these indirect effects. Second, these indirect and multiplier effects may be difficult to quantify because standard statistical methods could lead to incorrect results (see, e.g., Behrman et al. 2002; Montgomery and Casterline 1993).

In this study, we explore these indirect pathways by examining whether program effects diffuse both inside and outside the project area as well as what effect diffusion has on modern contraceptive use. Diffusion—the spread of ideas as evidenced by the adoption of behaviors related to those ideas—occurs through interactions at the interpersonal level or through impersonal channels, such as mass media (Measurement, Learning, and Evaluation Project 2010). Montgomery and Casterline (1996) and Kohler et al. (2001) hypothesized that diffusion can affect contraceptive use through two mechanisms: social learning and social influence. Social learning involves women learning through social interaction about other women’s experiences regarding, for instance, contraceptive side effects. This social learning may affect their probability of adopting a contraceptive method. Social influence makes particular behaviors normative, which in turn makes them easier for an individual to adopt.

We provide evidence on diffusion of a FP program in two states in Nigeria. The program was mainly implemented in the capital city of each state with the goal of increasing modern contraceptive use, particularly among the urban poor. Although the program was focused on the two capital cities, we have data representative of the entire states, including a five-year contraceptive calendar, which allows us to conduct two separate analyses. First, we determine whether any program effects spread beyond the two targeted intervention cities. Second, we use the calendar data to estimate dynamic models that examine the effects of lagged community-level modern contraceptive use (a variable often used as a proxy for social learning) on individual contraceptive behavior, controlling for individual-level characteristics including the individual’s lagged modern contraceptive use.

## Background

### Diffusion

Montgomery and Casterline (1996:151) noted that “diffusion can retard or accelerate fertility declines, can shape patterns of contraceptive method choice, and can either frustrate or facilitate the efforts of family planning programs.” The authors outlined the theoretical constructs of social learning and social influence and developed a statistical model, but the study lacked empirical validation.

In work that followed, Kohler et al. (2000, 2001) and Behrman et al. (2002) tested versions of Montgomery and Casterline’s model on modern contraceptive use. Kohler et al. (2000) used Kenya Demographic and Health Survey (KDHS) data to develop measures of social interactions based on KDHS sample clusters, which could lead to noisy indicators because the entire cluster may not be representative of a respondent’s social group. In addition, the analysis was limited because the data are cross-sectional, and the authors were unable to control for the potential endogeneity of the social interaction variables due to a lack of instrumental variables. Behrman et al. (2002), using longitudinal data, applied fixed-effects methods to correct for bias in the social interaction measures. They found that standard methods overstate the effect of social interactions but still found positive evidence of diffusion using fixed effects.

Kohler et al. (2001) tested for diffusion effects through the avenue of mass communication. They calculated the average number of women in a community, excluding the index woman, who recalled hearing a FP message on the radio. By excluding the respondent’s response, the variable is less likely to be contaminated by selective recall or acquiescence bias by the index respondent. This variable had a positive effect on ever use of modern contraception.

More recent studies on social influence or community effects have mainly focused on African countries. Elfstrom and Stephenson (2012) found positive effects for community-level factors, such as fertility and gender norms, on modern contraceptive use in 11 African countries. Stephenson et al. (2008) focused on the community climate for female autonomy and found few significant effects on modern contraceptive use. In Ethiopia, Alvergne et al. (2011) examined the role of person-to-person contact through either friendship or spatial networks on modern contraceptive use and found stronger marginal effects relative to individual sociodemographic characteristics, whereas Mace and Colleran (2009) found little evidence of kin influence on modern contraceptive uptake. Colleran and Mace (2015) used individual, social network, and community-level variables to study modern contraceptive use in Poland and found that social networks were more important than individual-level variables. Montgomery et al. (2001) used longitudinal data to measure the impact of diffusion on modern contraceptive use in Ghana. Diffusion indicators represent four mechanisms: mass media exposure, geographic mobility, contact with FP health workers, and social networks. When Montgomery et al. compared results of models using fixed-effect methods that took advantage of the longitudinal data with models that use cross-sectional methods, they found that cross-sectional methods overstated the impact of diffusion on modern contraceptive use but still found significant impacts of diffusion in correctly estimated longitudinal models.

Although individual-level longitudinal data have substantial utility in measuring diffusion effects, they are frequently not available. However, two studies used data that are longitudinal at the community level to great advantage. Montgomery and Casterline (1993) and Rosero-Bixby and Casterline (1994) applied similar approaches in Taiwan and Costa Rica, respectively. The outcomes are a measure of marital fertility in Taiwan and fertility regulation in Costa Rica. The community-level longitudinal data allowed them to estimate dynamic models in which the diffusion measure was the lagged value of the dependent variable. They then used a combination of fixed effects and instrumental variables methods to consistently estimate the model’s parameters. In both countries, the authors found significant diffusion effects. In Costa Rica, they also found diffusion effects across contiguous communities: communities in close proximity to communities with high modern contraceptive use are more likely to have increased future use. Our statistical methods are closely related to these studies except that we combine individual- and community-level data with a semiparametric maximum likelihood estimation strategy.

### Study Site and the Nigerian Context

Nigeria has low overall modern contraceptive use, estimated at 10% nationally among women whose marital status was in union in the 2013 Nigeria Demographic and Health Survey (National Population Commission (NPC) [Nigeria] and ICF International (2014); Nigeria Demographic and Health Survey 2013). Use is higher in urban areas (17%) than rural areas (6%) and varies by geographic zone of the country, with highest use in the southwest (38%) and lowest use in the northeast (3%) and northwest (4%). This study focuses on two study sites providing different contexts: Kaduna State in the northwest and Oyo State in the southwest to examine diffusion of program activities in Kaduna City (capital of Kaduna State) to other urban and rural areas of the state and likewise from Ibadan (capital of Oyo State) to other urban and rural areas of Oyo State. Based on the 2013 DHS (2014), Kaduna State had a lower total fertility rate (TFR) than Oyo State (4.1 vs. 4.5); however, among women aged 40–49, women in Kaduna State had more children ever born than women in Oyo (5.7 vs. 5.1). Women in Kaduna State marry earlier than women in Oyo State (median age 17.3 vs. 20.3 years), and the 2013 modern contraceptive prevalence rate (mCPR) among women in union is lower in Kaduna State than in Oyo State (18.5 vs. 24.4), although both states have higher mCPR than Nigeria as a whole.

We describe our data in the next section. However, here we provide additional context based on our 2015 data. Data from our study disaggregated at the capital city (Kaduna City and Ibadan), other urban areas, and rural areas demonstrate distinctions between them (Table 1). In Kaduna State, the primary language spoken is Hausa. In urban areas of the state, the majority of the population is Muslim; in rural areas, the population is about one-half Muslim and one-half Christian. In Oyo State, the majority of the population speaks Yoruba. In urban areas of Oyo State, about one-half of the population is Muslim, and one-half is Christian. In rural areas, a greater percentage of the population is Muslim.

**Table 1:** Descriptive statistics for Kaduna and Oyo State samples by capital city, other urban areas, and rural residence: Nigeria, 2015

	Kaduna State						Oyo State					
	Kaduna City		Other Urban Areas (noncapital)		Rural Areas		Ibadan City		Other Urban Areas (noncapital)		Rural Areas	
	Mean	SD	Mean	SD	Mean	SD	Mean	SD	Mean	SD	Mean	SD
Modern Contraceptive Use (%)	0.21	0.41	0.10	0.30	0.16	0.36	0.36	0.48	0.31	0.46	0.10	0.30
Age (%)												
15–19	0.21	0.40	0.22	0.41	0.23	0.42	0.16	0.37	0.20	0.40	0.19	0.40
20–24	0.18	0.38	0.20	0.40	0.19	0.39	0.15	0.36	0.15	0.36	0.21	0.41
25–29	0.19	0.39	0.18	0.38	0.16	0.37	0.18	0.39	0.15	0.36	0.19	0.39
30–34	0.15	0.36	0.15	0.35	0.14	0.35	0.18	0.39	0.15	0.36	0.14	0.35
35–39	0.13	0.33	0.11	0.31	0.12	0.32	0.17	0.38	0.13	0.33	0.11	0.31
40–44	0.07	0.25	0.08	0.28	0.09	0.29	0.10	0.30	0.13	0.33	0.07	0.26
45–49	0.09	0.28	0.07	0.26	0.07	0.26	0.07	0.25	0.09	0.29	0.07	0.26
Education (%)												
None	0.13	0.33	0.52	0.50	0.42	0.49	0.01	0.11	0.05	0.23	0.56	0.50
Primary	0.13	0.34	0.15	0.35	0.23	0.42	0.16	0.36	0.17	0.37	0.23	0.42
Junior secondary	0.14	0.35	0.09	0.30	0.14	0.35	0.16	0.37	0.12	0.32	0.07	0.25
Senior secondary	0.40	0.49	0.14	0.34	0.19	0.39	0.46	0.50	0.44	0.50	0.12	0.33
Higher	0.20	0.40	0.11	0.32	0.02	0.13	0.21	0.40	0.22	0.42	0.02	0.13
Marital Status (%)												
Never married	0.34	0.47	0.15	0.36	0.13	0.34	0.25	0.44	0.30	0.46	0.14	0.35
In union	0.61	0.49	0.83	0.38	0.83	0.38	0.70	0.46	0.66	0.47	0.84	0.37
Divorced/widowed	0.05	0.23	0.02	0.14	0.04	0.19	0.05	0.22	0.04	0.19	0.01	0.12
Religion (%)												
Christian	0.36	0.48	0.17	0.37	0.54	0.50	0.53	0.50	0.46	0.50	0.36	0.48
Muslim	0.64	0.48	0.83	0.37	0.46	0.50	0.48	0.50	0.54	0.50	0.64	0.48
Primary Language Spoken at Home (%)												
Hausa	0.76	0.43	0.94	0.24	0.98	0.13	0		0		0.00	0.06
Yoruba	0.00	0.02	0.00	0.04	0.00	0.05	0.87	0.34	0.92	0.28	0.98	0.14
English/Pigeon English	0.24	0.43	0.06	0.23	0.02	0.12	0.13	0.33	0.08	0.28	0.01	0.09
Other	0.00	0.02	0		0		0.00	0.04	0		0.01	0.09
Wealth (%)												
Poorest	0.01	0.08	0.16	0.37	0.34	0.47	0.00	0.04	0.02	0.13	0.60	0.49
Poor	0.02	0.14	0.26	0.44	0.37	0.48	0.01	0.11	0.06	0.23	0.19	0.39
Middle	0.13	0.33	0.21	0.41	0.24	0.43	0.15	0.36	0.22	0.42	0.14	0.35
Rich	0.37	0.48	0.23	0.42	0.04	0.19	0.42	0.49	0.34	0.47	0.06	0.23
Richest	0.48	0.50	0.14	0.34	0.01	0.11	0.41	0.49	0.37	0.48	0.01	0.10
NURHI Program Variables (%)												
Outreach	0.26	0.44	0.53	0.50	0.38	0.49	0.21	0.41	0.21	0.41	0.09	0.28
Radio	0.48	0.50	0.60	0.49	0.34	0.48	0.92	0.28	0.76	0.43	0.25	0.43
TV	0.39	0.49	0.11	0.32	0.03	0.17	0.59	0.49	0.41	0.49	0.06	0.24
NURHI Badge	0.10	0.30	0.08	0.25	0.03	0.17	0.36	0.48	0.19	0.40	0.05	0.21
Traveled to NURHI Program City (%)	0.07	0.26	0.07	0.25	0.08	0.26	0.02	0.15	0.09	0.28	0.07	0.26
Praise (% yes)	0.35	0.48	0.49	0.50	0.46	0.50	0.42	0.49	0.54	0.50	0.41	0.49
Sample Size (*N*)	2,801		1,124		1,706		720		1,121		629	

**Fig. 1:**
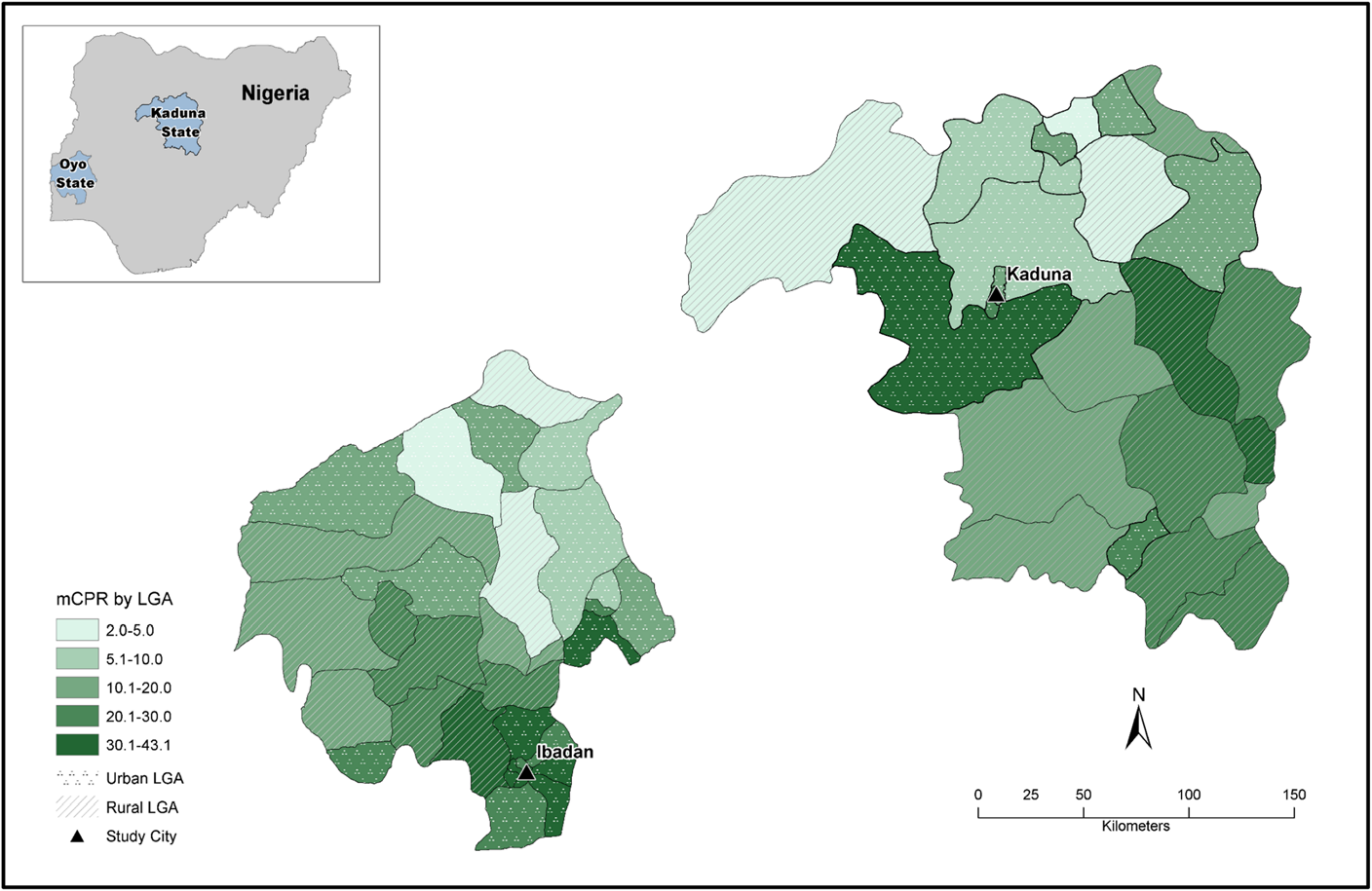
Map of modern contraceptive prevalence rate by local government area in Oyo and Kaduna States

At the time of the survey, modern contraceptive use differed by state (Table 1 and Fig. 1). About one-third of all women in urban areas of Oyo State reported using modern contraception. Use was lower in urban areas in Kaduna State. Conversely, a greater percentage of all women in rural Kaduna State reported using modern contraception compared with women in rural areas of Oyo State. Modern contraceptive use was higher in the capital cities than other urban areas in both states. In previous analyses using these data, we found that Muslims are less likely to use contraception; married women, older women, and more educated women are more likely to use contraception (Measurement, Learning, and Evaluation Project Nigeria Team 2017).

Figure 1 presents the distribution of mCPR by local government area (LGA) in Oyo and Kaduna States. In Oyo State, LGAs surrounding Ibadan City have higher contraceptive use than most of the rural and urban LGAs that are farther away. In Kaduna State, Kaduna City is surrounded both by LGAs with higher contraceptive use in the area and by LGAs with very low contraceptive use; this likely reflects the fact that the northern parts of the state are more Muslim compared with the southern parts of the state. High mCPR, however, does not necessarily translate into lower fertility (see Bledsoe et al. 1994; Caldwell and Caldwell 1987).

## Data Set and Descriptive Statistics

The Nigerian Urban Reproductive Health Initiative (NURHI) sought to increase modern contraceptive use through a variety of demand- and supply-side activities implemented in six cities in Nigeria (Abuja, Benin City, Ibadan, Ilorin, Kaduna, and Zaria) from 2011 to 2015 (see http://www.nurhi.org/ for NURHI program details). In 2015, as part of endline data collection, the MLE Project conducted a cross-sectional survey in two states in Nigeria to determine whether NURHI program elements diffused from implementation cities to other urban or rural areas.

The MLE Project collected state-level data from households and women using a multistage sampling design. First, the 2006 Nigerian Population and Housing Census frame was used to randomly sample clusters from Ibadan and Kaduna cities where NURHI was implemented, and clusters from other urban and rural areas across Kaduna and Oyo States, and then a representative sample of households was selected from the clusters. All eligible women aged 15–49 were invited to complete an interviewer-administered paper-and-pencil survey after providing informed consent. The survey took about 1–1.5 hours to complete. Of the 8,101 surveyed participants, 21.8% lived in rural areas, 21.0% lived in other urban areas outside Ibadan and Kaduna City, and 57.2% lived in the two capital cities. The study protocol and consent forms were approved by the Institutional Review Board at the University of North Carolina at Chapel Hill and by the National Health Research Ethics Committee of Nigeria in Nigeria.

Data collected included individual-level sociodemographic information, whether the respondent was currently using modern contraception,^1^ and exposure to NURHI program elements. Although the NURHI program had a large number of components, we use four measures that were important in the original six NURHI program cities (Measurement, Learning, and Evaluation Project Nigeria Team 2017): exposure to NURHI FP messages on radio programs; saw NURHI FP messages on television (TV); saw a provider wearing a badge clearly identifying a NURHI-trained provider (hereafter referred to as *NURHI badge*); and exposure to FP messages at weddings, naming ceremonies, freedom ceremonies, school graduation ceremonies, or at Christmas/Eid celebrations (these were NURHI-organized outreach activities and are referred to *NURHI outreach* hereafter).^2^ An additional diffusion-related measure included was whether the respondent had traveled to one of the six NURHI program cities in the last year. We group this variable with the program exposure variables. In addition, one goal of NURHI’s efforts was to improve attitudes and perceptions about modern contraceptive use. To capture changing attitudes, we include a measure of whether the respondent feels she would receive praise for using modern contraception. Given that a key approach of the NURHI program was to change ideation (i.e., values, myths, and communication) around FP use, this praise variable is a way to capture a key component of the NURHI program strategy, and we use it as a proxy for social influence.^3^ Finally, retrospective information about modern contraceptive use was captured using a five-year contraceptive calendar ending at the time of the survey. Specific questions used to define important variables are available in the online appendix.

Following Kohler et al. (2000), we construct community-level program exposure variables using study clusters as a proxy for community, where we drop the index woman when calculating the average to remove individual-level bias from the community-level exposure variable.^4^ This may be a better variable than individual recall: it is well known that individual-level recall of exposure is subject to measurement error, and averaging reduces these effects (see Chowdhury and Nickell 1985; Swaffield 2001).

This analysis examines data at varying levels: jointly for the two cities, for rural areas of the two states, and for noncapital cities of the two states. Table 1 presents data disaggregated by state and site type. As one might expect, modern contraceptive use is substantially higher in other urban areas compared with rural areas in Oyo State but was unexpectedly lower in other urban areas compared with rural areas in Kaduna State. The age distributions are similar in the disaggregated samples. However, the urban samples are generally more highly educated and wealthier than the rural samples. In Oyo State, the percentage of women in union is higher in rural areas than the urban sites. In Kaduna State, Kaduna City has the lowest percentage of women in union, and other urban and rural samples are similar.

We also conduct an analysis using data for the entirety of both states (i.e., data from the cities of Ibadan and Kaduna and all other areas of the two states). A retrospective contraceptive calendar was used to generate a binary indicator for whether the respondent used modern contraception at any point in each of the five preceding years. These individual-level, time-varying variables are used to create the percentage of respondents in each community using modern contraceptives at each time point, excluding the index respondent. The lagged percentage of women in her community using modern contraception captures the effect of social learning on individual use. Following Montgomery and Casterline (1993), we create a variable for lagged contraceptive use in nearby communities as an additional avenue for social learning. The nearby community contraceptive use variable is generated using the buffer tool in ArcGIS v10.0. Distance bands ranging from 5 to 50 kilometers were placed around each index community, and community-level contraceptive use is measured for all other surveyed communities within each distance band. Community-level modern contraceptive use is inversely weighted by distance so that contraceptive use in closer areas was weighted more heavily. A 10-kilometer band is used in the multivariate analysis because preliminary analysis revealed that it had the largest effect among various distances. Some communities in the sample had no surveyed community within 10 kilometers, so we set this variable to 0 for these communities and include a dummy variable indicating that there was no neighboring surveyed community. This was true for 21% of respondents.

To capture social influence in the pseudo-longitudinal data model, we use the community-level praise variable. Because this variable cannot be backdated, we have to either assume that it did not change much over the five-year period—which is highly unlikely given the FP promotions ongoing during this period—or just allow it to have an effect in the final year. We try both alternatives and report the results for using the Year 5 value for all years, given that the results are similar.

## Statistical Methods

### Cross-Sectional Model

We estimate cross-sectional models to study diffusion effects of mass communication in rural and other urban areas outside the program cities because relevant information is retrospectively available at only one time point (exposure in the last year or last three months). We estimate three models. The first model is

1 $$ \ln \left[\frac{P\left({C}_{ij}=1\right)}{P\left({C}_{ij}=0\right)}\right]={\boldsymbol{\upalpha}}_1{\mathbf{Com}}_j+{\boldsymbol{\upalpha}}_2{\mathbf{E}}_{ij}+{\boldsymbol{\upalpha}}_3{\mathbf{X}}_{ij}+{\upmu}_j, $$

where the dependent variable is the log odds that woman *i* (*i* = 1,2, . . . , *N*_*j*_) from community *j* (*j* = 1,2, . . . , *M*) is currently using modern contraception. **Com**_*j*_ represents community-level variables, such as the dummy indicator that the observation is from a community in Oyo State (Kaduna State is reference); **E**_*ij*_ represents individual-level exposure variables, such as recall of having heard a NURHI radio program; **X**_*ij*_ represents individual-level control variables, such as age and education; and μ_*j*_ represents unobserved community-level factors that could influence use, such as the degree of social heterogeneity in the community. Even if the μs are random across communities, the fact that they do not vary within a community means that the standard errors estimated by simple logit must be adjusted for clustering or that the random-effects maximum likelihood estimator must be used. We use the maximum likelihood estimator so that we can estimate the variance of μ.

In the second model, we replace individual-level exposure, **E**_*ij*_, in Eq. (1) with ${\overset{\sim }{\mathbf{E}}}_{ij}$, which represents average community-level NURHI exposure associated with woman *i* from community *j* with woman *i* excluded. The non–self-average variable should not be correlated with the individual-level error, so recall bias should be mitigated; the averaging may also mitigate the effects of measurement error. However, there could still be bias if there is correlation between ${\overset{\sim }{\mathbf{E}}}_{ij}$ and μ_*j*_, which could occur, for example, if program activities are targeted to some communities.

**Fig. 2:**
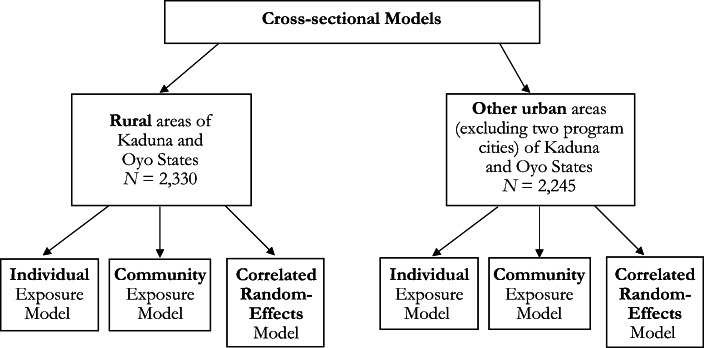
Summary of the estimation methods for the cross-sectional models in rural and other urban areas of Kaduna and Oyo States. Individual exposure model: Diffusion-related variables are the individual’s recall of program variables with no correction for recall or acquiescent bias. Community exposure model: Diffusion-related variables are community averages excluding the target individual. The method corrects for bias at the individual level but not at the community level. Correlated random*-*effects model: Diffusion-related variables are the individual’s recall of program variables with a correction for recall or acquiescent bias at the community level but not at the individual level.

Finally, to address this possible correlation between included variables and the community-level error, we estimate a correlated random-effects model (see Schunck 2013; Schunck and Perales 2017; Wooldridge 2009) of the following form:

2 $$ \ln \left[\frac{P\left({C}_{ij}=1\right)}{P\left({C}_{ij}=0\right)}\right]={\boldsymbol{\upalpha}}_1{\mathbf{Com}}_j+{\boldsymbol{\upalpha}}_2\left({\mathbf{E}}_{ij}-{\overline{\mathbf{E}}}_j\right)+{\boldsymbol{\upalpha}}_3\left({\mathbf{X}}_{ij}-{\overline{\mathbf{X}}}_j\right)+{\boldsymbol{\uplambda}}_1{\overline{\mathbf{E}}}_j+{\boldsymbol{\uplambda}}_2{\overline{\mathbf{X}}}_j+{\upmu}_j, $$

where ${\overline{\mathbf{X}}}_j$ and ${\overline{\mathbf{E}}}_j$ denote community averages of the **X** and **E** variables, respectively. In the linear case, this is referred to as the Mundlak model (Mundlak 1978), and it is well known that the estimates of **α**_2_ and **α**_3_ will be identical to the fixed-effects estimator that includes community dummy variables. A joint test of the null hypothesis that **α**_2_ = **λ**_1_ and **α**_3_ = **λ**_2_ is a robust version of the Hausman test and is equivalent to a test of the null hypothesis of no correlation between **X** and **E** and μ but not **Com**_*j*_. The pros and cons of the three methods are summarized in Fig. 2.

### Pseudo-Longitudinal Data Model

**Fig. 3:**
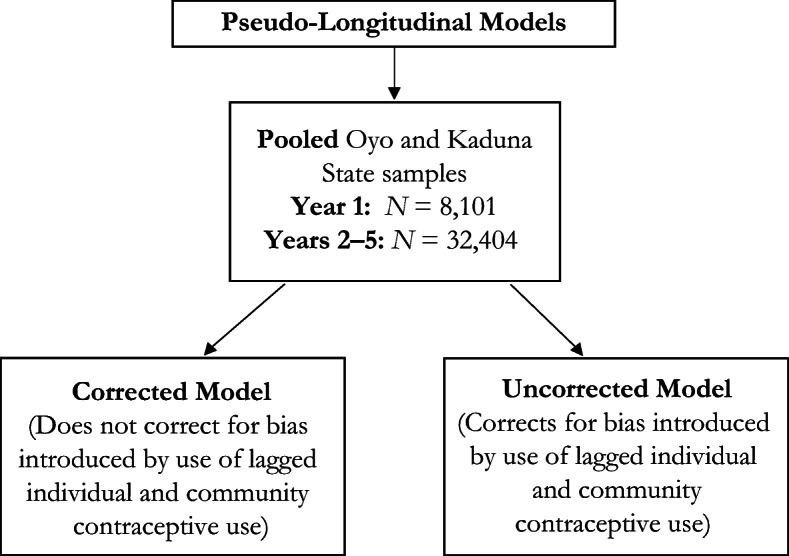
Summary of pseudo-longitudinal data model using pooled sample from Kaduna and Oyo States

Our second analysis uses a pseudo-panel data set where we have five observations for each woman spaced one year apart (Table 2 and Fig. 3). The primary outcome is whether the respondent used modern contraception at any time during each of the five periods. The primary explanatory variables are two proxies for social learning: modern contraceptive use (removing the index woman) in the respondent’s community lagged by one year, and modern contraceptive use in the respondent’s surrounding communities lagged by one year. We also include one explanatory variable for social influence: the proportion of women (removing the index woman) who responded that they would receive praise for using modern contraception.

**Table 2:** Descriptive statistics for the pseudo-longitudinal data for entire Kaduna and Oyo States

	Year 1	Year 2	Year 3	Year 4	Year 5
mCPR	0.15	0.19	0.21	0.23	0.26
	(0.36)	(0.39)	(0.40)	(0.42)	(0.44)
Age (years)	23.99	24.99	25.99	26.99	27.99
	(9.48)	(9.48)	(9.48)	(9.48)	(9.48)
Age, Squared	665.44	714.42	765.40	818.38	873.36
	(487.35)	(516.02)	(534.70)	(553.41)	(572.13)
Education					
None	0.26	0.26	0.26	0.26	0.26
	(0.44)	(0.44)	(0.44)	(0.44)	(0.44)
Primary	0.24	0.25	0.25	0.23	0.19
	(0.42)	(0.43)	(0.43)	(0.42)	(0.39)
Junior secondary	0.13	0.14	0.15	0.15	0.17
	(0.34)	(0.35)	(0.36)	(0.36)	(0.37)
Senior secondary	0.21	0.22	0.23	0.24	0.26
	(0.41)	(0.41)	(0.42)	(0.43)	(0.44)
Higher	0.10	0.11	0.11	0.12	0.13
	(0.30)	(0.31)	(0.32)	(0.33)	(0.33)
In Union	0.61	0.64	0.67	0.70	0.72
	(0.49)	(0.48)	(0.47)	(0.46)	(0.45)
Oyo, Rural					0.08
					(0.27)
Kaduna, Rural					0.21
					(0.41)
Oyo, Other Urban					0.14
					(0.35)
Kaduna, Other Urban					0.14
					(0.35)
Kaduna					0.35
					(0.48)
Ibadan					0.09
					(0.28)
Always a Resident					0.50
					(0.50)
No Neighboring Community in Sample					0.21
					(0.41)
Community mCPR	0.18	0.20	0.23	0.25	0.28
	(0.12)	(0.14)	(0.15)	(0.16)	(0.17)
Neighboring Community mCPR	0.17	0.20	0.23	0.25	0.28
	(0.12)	(0.13)	(0.15)	(0.16)	(0.17)
Praise					0.44
					(0.24)
Sample Size	8,101	8,101	8,101	8,101	8,101

*Note:* Standard deviations are shown in parentheses.

Following Montgomery and Casterline (1993), we specify a dynamic model. The key outcome used by Montgomery and Casterline (1993) was community-level contraceptive use, which was assumed to be influenced by past use in the same community plus past use in surrounding communities. Because we observe our key outcome for each woman at each time point, we can measure the effects of social learning and social influence on the individual respondent’s behavior with a control for the woman’s own lagged contraceptive use. In addition, rather than using first differences coupled with instrumental variables to correct for endogenous explanatory variables, which has a very high cost in terms of lost degrees of freedom, we use semiparametric maximum likelihood estimation. This requires us to estimate a separate equation for contraceptive use at Year 1 to correct for the endogeneity of lagged contraceptive use in the dynamic model.

The equation of primary interest is

3 $$ \ln \left[\frac{P\left({C}_{tij}=1\right)}{P\left({C}_{tij}=0\right)}\right]={\upalpha}_1^C{C}_{t\kern.3em -1, ij}+{\upalpha}_2^C{\overset{\sim }{Con}}_{t-1, ij}+{\upalpha}_3^C Con{10}_{t-1,j}+{\upalpha}_4^C{\overset{\sim }{P}}_{tij}+{\boldsymbol{\upbeta}}^c{\mathbf{X}}_{tij}+{\upmu}_j^C+{v}_{ij}^C, $$

where the dependent variable is the log odds that woman *i* (*i* = 1,2, . . . , *N*_*j*_) from community *j* (*j* = 1,2, . . . , *M*) at year *t* (*t* = 2,3,4,5) is using modern contraception. Year 1 data are used to create the lagged variables. The explanatory variables are whether the respondent used a modern method in the previous year (*C*_*t* – 1, *ij*_), modern contraceptive use in the respondent’s community excluding the index respondent (${\overset{\sim }{Con}}_{t-1, ij}$), modern contraceptive use in the respondent’s surrounding communities in the previous year (*Con*10_*t* – 1, *ij*_), the proportion of women in the community who believe that they will receive praise for using modern contraception excluding the index respondent (*P*_*tij*_, with the *t* subscript indicating that we zero out this variable in Years 1–4 in one of the specifications and simply use the Year 5 value for all years in an alternative specification), and a vector of control variables, such as age and education. The α^*C*^ are scalar parameters to be estimated, and **β**^*c*^ is a vector of parameters to be estimated.

We specify a multicomponent error term. ${\upmu}_j^C$ represents time-invariant unobservable variables associated with community *j* that could influence a respondent’s contraceptive use decision—for example, the level of social heterogeneity in the community. ${v}_{ij}^C$ represents time-invariant unobservable variables for respondent *i* from community *j* that could influence the respondent’s decision—for example, a woman’s perception of how many of her children will survive childhood. Implicit in the logistic specification is an individual-level time-varying error.

As discussed later, Eq. (3) should be estimated jointly with equations for the two social learning variables (${\overset{\sim }{Con}}_{t-1, ij}$ and *Con*10_*t* – 1, *ij*_) and the social influence variable (*P*_*tij*_) to control for possible correlation between these three variables and the components of the error term in Eq. (4). Unfortunately, equations for *Con*10_*t* – 1, *ij*_ and *P*_*tij*_ were weakly identified and caused a great deal of instability when they were jointly estimated with Eq. (3). Therefore, we retain these two variables in Eq. (3) when we estimate this equation by itself with no correction for possible bias but drop them from the joint estimation. Joint estimation therefore involves Eq. (3) and the two equations that follow.

The equation for community-level contraceptive use is

4 $$ {\overset{\sim }{Con}}_{tij}={\boldsymbol{\upbeta}}^{Con}{\mathbf{X}}_{tj}^{Con}+{\upmu}_j^{Con}+{v}_{ij}^{Con}+{\upvarepsilon}_{tij}^{Con}, $$

where the dependent variable is the proportion of women using modern contraception from community *j* at year *t* (*t* = 1,2,3,4) with respondent *i* omitted, which is why we still have three subscripts on the dependent variable. We treat this variable as continuous. The **X**s represent community averages for the explanatory variables from Eq. (3), ${\upmu}_j^{Con}$ represents time-invariant community-level variables that affect community-level use, ${v}_{ij}^{Con}$ is a time-invariant individual-level error term, and ${\upvarepsilon}_{tij}^{Con}$ is a time-varying error term that is assumed to follow a normal distribution with a mean of 0 and a constant variance. Finally, we must control for one initial condition: the individual’s decision to use modern contraception at Year 1, as shown in Eq. (5).

5 $$ \ln \left[\frac{P\left({C}_{1 ij}=1\right)}{P\left({C}_{1 tj}=0\right)}\right]={\boldsymbol{\upbeta}}^{C1}{\mathbf{X}}_{1 ij}+{\upmu}_j^{C1}+{v}_{ij}^{C1}. $$

The dependent variable is the log odds that individual *i* from community *j* is using modern contraception in Year 1. The **X** are the same set of variables as Eq. (3) but just for Year 1.

We assume that community-level unobservable characteristics (μs) in Eqs. (3)–(5) are correlated because there could be common unobservable variables that affect all outcomes. Furthermore, we assume that the individual-level time-invariant errors (*v*s) in Eqs. (3), (4), and (5) are also correlated. The implication of these assumptions is that the estimation of Eq. (3) by simple logit will lead to biased parameter estimates.

Our modeling strategy involves joint estimation of the three equations using semiparametric maximum likelihood. Joint estimation controls for the endogeneity of the initial use of modern contraception and our measure for social learning (${\overset{\sim }{Con}}_{t-1, ij}$). Rather than make a specific parametric assumption about the distribution of the μs and *v*s (for example, multivariate normality is often assumed), we use a variation of the discrete factor approximation (Heckman and Singer 1984). Specifically, we use what Mroz (1999) refers to as *nonlinear heterogeneity*, where mass points are estimated for each equation along with a common set of probabilities. This form for the discrete factor model allows for very general patterns of correlation and has been shown to work very well in Monte Carlo experiments (Guilkey and Lance 2014; Mroz 1999). Many of the regressors are time-varying, and this is the main source of identification for the model (Bhargava 1991). For example, only Year 1 exogenous variables are assumed to affect modern contraceptive use at Year 1.

## Results

### Cross-Sectional Results

Table 1 provides information on NURHI program exposure in all areas of each state. Interestingly, and related to diffusion of FP programming, the other urban sample from Kaduna has higher reported exposure to outreach and radio programming than the Kaduna City sample even though the program was focused on Kaduna City. Additionally, reported outreach exposure was higher in rural areas of Kaduna State than in Kaduna City. This high reported outreach outside Kaduna City may reflect people traveling to the capital city and being exposed or a misunderstanding of the outreach question that was asking about FP messages at specific life events, such as weddings and freedom ceremonies, and not simply FP discussions at these events. Therefore, results for the outreach variable should be interpreted cautiously. As expected, the pattern for Oyo State shows higher NURHI exposure in Ibadan followed by other urban areas, with rural areas being the lowest. The percentage who say that they would receive praise for using family planning is higher in other urban areas than the other areas in both states. The percentage of respondents who traveled to one of the program cities is similar across locations in Kaduna State and highest in other urban areas in Oyo State. We do not report community average means for the exposure variables because these averages are almost identical to reported individual-level averages.

Table 2 provides descriptive statistics for variables in the pseudo-longitudinal sample for each of the five years separately, with the Year 1 column containing measures backdated five years and the other columns defined similarly. For variables that are not time-varying, we present the mean and standard deviation in the Year 5 column when they were measured. We see that modern contraceptive use in both states increased steadily over time from about 15% to 26%. The average age of respondents was about 28 at the time of the survey, which backdates to an average of about 24 years in the first period. There is some time variation in education level because we construct this variable based on the respondent’s age at the time of the survey and then assume normal progression through the grades until the woman reached her terminal level. The percentage of respondents who were in union over the last five years demonstrates increasing union status over time, as expected. Further, community average modern contraceptive use (excluding each respondent in turn) and neighboring community modern contraceptive use show similar trends to those for individual-level contraceptive use over time. Also presented in Table 2 are statistics for the praise variable in Year 5 when it was measured.

Table 3 presents the estimation results for the control variables used in the models separately for rural and other urban areas. We present results for only the individual exposure model (see Fig. 2) because the results for the other models were quite similar.

**Table 3:** Cross-sectional model results for modern contraceptive use for rural and other urban Kaduna and Oyo States: Control variables

	Rural Model				Other Urban Model			
	Coef.	SE	Margin	SE Margin	Coef.	SE	Margin	SE Margin
Oyo State	–0.820	1.068	–0.074	0.088	1.708**	0.549	0.218	0.068
Age (years) (ref. = 15–19)								
20–24	0.720*	0.316	0.080	0.039	1.768***	0.317	0.250	0.044
25–29	1.304***	0.313	0.159	0.043	1.994***	0.331	0.290	0.047
30–34	1.140***	0.323	0.137	0.045	2.055***	0.338	0.304	0.049
35–39	1.398***	0.325	0.178	0.048	2.154***	0.345	0.327	0.052
40–44	1.200**	0.346	0.151	0.051	2.158***	0.347	0.332	0.053
45–49	0.761*	0.378	0.089	0.050	1.835***	0.366	0.279	0.058
Education (ref. = none)								
Primary	0.543**	0.202	0.058	0.023	0.640**	0.235	0.087	0.034
Junior secondary	0.497*	0.253	0.055	0.030	0.506^†^	0.288	0.069	0.042
Senior secondary	0.503*	0.249	0.055	0.029	0.583*	0.245	0.077	0.034
Higher	1.209*	0.487	0.158	0.077	0.779**	0.269	0.108	0.040
In Union	1.119***	0.277	0.090	0.017	0.534**	0.189	0.064	0.021
Muslim	–1.243***	0.206	–0.123	0.020	–0.299*	0.145	–0.039	0.019
Primary Language Spoken at Home (ref. = Hausa)								
Yoruba	0.772	1.062	0.086	0.129	–0.818	0.550	–0.100	0.064
English	0.554	0.557	0.064	0.072	–1.068*	0.482	–0.111	0.039
Wealth (ref. = richest)								
Poorest	1.252^†^	0.751	0.135	0.085	–0.619	0.384	–0.071	0.039
Poor	1.455^†^	0.745	0.166	0.094	–0.639*	0.299	–0.074	0.031
Middle	1.823*	0.738	0.231	0.109	0.021	0.200	0.003	0.026
Rich	1.553*	0.741	0.211	0.123	0.226	0.166	0.029	0.022
σ_μ_	0.466***	0.106			0.495***	0.103		
Sample Size	2,330				2,245			

*Note:* Coefficients presented are from individual exposure model but are representative of all three models.

†*p* < .10; *p < .05; **p < .01; ***p < .001

Table 4 presents results for the exposure variables for all three models for both samples, and Table 5 presents the results for six estimations (the three models and the two samples), where we replace the exposure variables with the praise variable. Results for the control variables are available in the online appendix, Table A1. All tables present coefficient estimates with standard errors, the average marginal effect, and the standard error of the average marginal effect. We also indicate whether the coefficient is significantly different from 0 at four significance levels (.10, .05, .01 and .001). Given that we are estimating logit models, the coefficients can be compared only for sign and significance because of possible differences in scaling. However, the average marginal effects can be directly compared. So, for example, the average marginal effect for individual radio exposure in the rural model (Table 4) is 0.030, which means going from no exposure to exposure results in a 3 percentage point increase in predicted modern contraceptive use.

**Table 4:** Cross-sectional model results, modern contraceptive use for rural and other urban Kaduna and Oyo States: Exposure variable results for three estimation methods

	Model a: Individual Exposure				Model b: Community Exposure				Model c: Correlated Random Effects			
	Coef.	SE	Margin	SE Margin	Coef.	SE	Margin	SE Margin	Coef.	SE	Margin	SE Margin
Rural Model Exposures												
Radio	0.292^†^	0.151	0.030	0.016	1.494**	0.527	0.151	0.053	0.170	0.157	0.017	0.016
Outreach	0.526***	0.150	0.056	0.017	0.469	0.553	0.047	0.056	0.554***	0.158	0.057	0.017
NURHI Badge	–0.233	0.326	–0.022	0.029	1.860	1.529	0.188	0.154	–0.282	0.332	–0.026	0.029
TV	–0.066	0.319	–0.007	0.031	0.102	1.445	0.010	0.146	–0.101	0.330	–0.010	0.031
Traveled to NURHI program city	0.367	0.235	0.040	0.028	0.580	1.076	0.059	0.109	0.362	0.245	0.039	0.028
Other Urban Model Exposures												
Radio	0.177	0.157	0.022	0.020	0.045	0.656	0.006	0.084	0.147	0.163	0.019	0.020
Outreach	–0.185	0.143	–0.023	0.018	–2.066**	0.695	–0.264	0.088	–0.053	0.146	–0.007	0.019
NURHI Badge	0.590***	0.164	0.082	0.024	0.149	0.667	0.019	0.085	0.540**	0.170	0.074	0.025
TV	–0.113	0.142	–0.014	0.018	1.230*	0.709	0.157	0.090	–0.150	0.145	–0.019	0.018
Traveled to NURHI program city	–0.001	0.189	0.000	0.024	–0.971	1.024	–0.124	0.130	0.083	0.193	0.011	0.025

*Notes:* Model a shows estimates for individual-level variables. Model b shows estimates for mean community-level variables and average marginal effects. Model c presents within estimates.

†*p* < .10; *p < .05; **p < .01; ***p < .001

**Table 5:** Cross-sectional model results, modern contraceptive use for both rural and other urban Kaduna and Oyo States: Effects of receiving praise for family planning use

	Model a: Individual-Level Praise				Model b: Community-Level Praise				Model c: Correlated Random Effects			
	Coef.	SE	Margin	SE Margin	Coef.	SE	Margin	SE Margin	Coef.	SE	Margin	SE Margin
Praise, Rural Models	0.503***	0.140	0.051	0.014	1.445	0.584	0.146	0.059	0.435**	0.142	0.044	0.014
Praise, Other Urban Models	0.466***	0.126	0.059	0.016	0.827	0.518	0.106	0.066	0.422**	0.130	0.054	0.016

*Notes:* Model a shows estimates for individual-level variables. Model b shows estimates for mean community-level variables and average marginal effects. Model c presents within estimates.

†*p* < .10; *p < .05; **p < .01; ***p < .001

For the control variable results for rural areas (left side of Table 3), all age groups are significantly more likely to use modern contraception relative to the youngest age group (age 15–19) at the 5% significance level. The effect of age is nonlinear, with the largest coefficient for the 35–39 age group and the two smallest coefficients for the 20–24 and 45–49 age groups. The results for education are also fairly standard, with the point estimates for all the education dummy variables relative to no education being positive and significant at the 5% level or higher; the largest effect is for the highest level of education. The language dummy variables are not significant, but the coefficients on the four estimated wealth dummy variables are of similar magnitude, showing positive and sometimes significant effects at the 10% level relative to the richest category. This counterintuitive result for wealth may be due to the fact there is little variation in wealth in rural areas. Finally, women in unions are more likely to use modern contraception, whereas Muslims are less likely.

The results from Table 3 for other urban areas (excluding Ibadan and Kaduna cities) tell a similar story except for state of residence: other urban residents from Oyo State are more likely to use contraception than other urban residents from Kaduna State. Nonlinear age effects peak at ages 35–39, the largest positive effect for education is for the most-educated respondents, and the effects of being in a union and Muslim have signs opposite to those for rural areas. The wealth dummy variables are generally not significant predictors.

We now turn to results for the diffusion-related variables in Table 4. Recall that the individual exposure model (Model a) does not correct for bias in the exposure variables due to correlation with either the community or individual-level error term. The community exposure model (Model b) corrects only for correlation with the individual error, and the correlated random-effects model (Model c) corrects for correlation with the community-level error but not the individual-level error (see Fig. 2 for a summary of differences). In addition, the correlated random-effects model allows us to use a robust version of the Hausman test to test the null hypothesis of bias at the community level. Not surprisingly, the null hypothesis is rejected at the 0.1% significance level for both the rural and other urban samples; this means that the correlation between the regressors and the unobserved community-level variables is introducing bias, and thus we do not report estimates for **λ**_1_ and **λ**_2_ from Eq. (2).

Results for the three rural exposure models (individual exposure, community exposure, and correlated random effects) are presented in Table 4. The NURHI badge and TV variables and the dummy variable for travel to another NURHI program city are not significantly different from 0 in any of the three model estimations, and point estimates are often of the unexpected sign. Conversely, recall of NURHI radio programming and exposure to NURHI outreach have positive effects in all three models, and the results are significant at the 10% or higher level for two of three models for each exposure. The radio results are significant for the individual and community-level models at the 10% and 1% levels of significance, respectively, and not significant for the correlated random-effects model. The average marginal effects for radio are not consistent across estimation methods. This is in contrast to NURHI outreach exposure, where the marginal effects are quite similar for all estimation methods, but the marginal effect for the community exposure model has a large standard error.

The results for the other urban sample models (individual exposure, community exposure, and correlated random effects) presented in Table 4 do not suggest diffusion effects from mass communication. The signs of the variables are not consistent across models, and few results approach significance except for the NURHI badge recall variable, which is positive for all three models and is significant at the 1% level in the individual and correlated random-effects models. However, both its coefficient and marginal effect have large standard errors in the community exposure model.

One of the primary objectives of the NURHI program was to change attitudes and perceptions around the use of modern FP methods. Therefore, in addition to separate exposure variables, we also estimate models in which we drop these individual exposure variables and instead include whether the woman thought that she would receive praise from other community members for using modern contraception. This variable could be considered a measure of social influence and a possible summary measure for the large number of NURHI activities designed to promote modern contraceptive use. In fact, when we estimate simple regressions with praise as a function of NURHI program components, most had positive associations with praise (results available in online appendix, Table A2). We report the results for this variable for all three estimation methods for both the pooled rural and other urban samples in Table 5. All models include the same set of control variables, and the results for these variables are similar to those reported in Table 3 and are thus not shown. Thus, all that is reported in Table 5 is the estimated effect of the praise variable on modern contraceptive use from the six regressions. Four of the six estimated coefficients are significant at the 5% level of significance or higher, and all six coefficients are positive. The estimated average marginal effects are quite consistent both across estimation methods and across the rural and other urban samples. However, the community-level praise average marginal effects (Model b) are larger with larger standard errors.

Given the robust strength of the praise results regardless of sample or estimation method, these results clearly provide evidence that NURHI program activities had a positive association with modern contraceptive use beyond the program cities, and studies focusing solely on the program cities have probably understated program effects.

### Pseudo-Longitudinal Data Results

Table 6 presents results for the estimations using the pseudo-panel data set for the pooled samples for Oyo and Kaduna States (see the summary of methods in Fig. 3). Both the uncorrected and corrected results are presented in Table 6 so that a side-by-side comparison can be made. As in the cross-sectional models, we present estimated coefficients and standard errors and indicators for significance at standard significance levels. We also report average marginal effects and corresponding standard errors. Given the fact that we are averaging over a very large sample size, the standard errors of the average marginal effects are quite small. The uncorrected results are estimated using simple logit with a cluster correction to the standard errors.

**Table 6:** Pseudo-longitudinal diffusion effects for modern contraceptive use for the pooled Kaduna and Oyo States

	Uncorrected Results				Corrected Results			
	Coef.	SE	Margin	SE Margin	Coef.	SE	Margin	SE Margin
Constant	–6.400***	0.280			–8.937***	0.580		
Age (years)	0.211***	0.019	0.016	0.001	0.343***	0.031	0.026	0.005
Age, Squared	–0.003***	0.000	0.000	0.000	–0.005***	0.000	–0.001	0.000
Education (ref. = none)								
Primary	0.243**	0.077	0.019	0.006	0.409**	0.124	0.041	0.013
Junior secondary	0.363***	0.084	0.029	0.007	0.646***	0.122	0.067	0.014
Senior secondary	0.558***	0.071	0.044	0.006	0.939***	0.117	0.984	0.016
Higher	0.441***	0.088	0.036	0.008	0.806***	0.140	0.086	0.018
In Union	0.794***	0.078	0.059	0.006	1.076***	0.114	0.100	0.015
Muslim	–0.325***	0.057	–0.025	0.005	–0.606***	0.088	–0.060	0.012
State (ref. = Kaduna, other urban)								
Oyo, rural	–0.048	0.122	–0.004	0.009	–0.302	0.184	–0.028	0.019
Kaduna, rural	0.183	0.118	0.014	0.009	–0.340*	0.160	–0.032	0.015
Oyo, other urban	0.287*	0.120	0.022	0.001	0.995***	0.157	0.109	0.025
Kaduna	0.184^†^	0.103	0.014	0.014	0.520***	0.128	0.051	0.015
Ibadan	0.301*	0.134	0.024	0.011	1.241***	0.208	0.142	0.033
Year (ref. = 2)								
3	–0.189**	0.063	–0.014	0.005	–0.075**	0.065	–0.007	0.006
4	–0.149*	0.065	–0.011	0.005	0.040	0.071	0.004	0.007
5	–0.242^†^	0.124	–0.018	0.009	0.259***	0.083	0.026	0.009
Always a Resident	0.019	0.044	0.001	0.002	0.103^†^	0.617	0.010	0.006
No Neighboring Community in Sample	–0.204^†^	0.107	–0.015	0.008				
Lagged mCPR	3.775***	0.065	0.633	0.010	2.518***	0.095	0.375	0.039
Lagged Community mCPR	2.280***	0.221	0.171	0.018	1.116*	0.494	0.109	0.047
Lagged Neighboring Community mCPR	0.032	0.361	0.002	0.028				
Community Praise	0.447^†^	0.233	0.034	0.018				

*Note:* Corrected results dropped equations for neighboring community use and praise because they were unstable and weakly identified.

†*p* < .10; *p < .05; **p < .01; ***p < .001

The control variables behave much as expected for both uncorrected and corrected models. Age has a nonlinear effect, with modern contraceptive use being an increasing function of age but at a decreasing rate. Higher levels of education are associated with greater modern contraceptive use, with the largest effect seen for senior secondary education. The in union and Muslim dummy variables have opposite effects in the expected directions. Note that age, education, in union, and Muslim are all significant at the 0.1% level, and all except the Muslim dummy variable are time-varying variables. This is important for the corrected model that relies on time variation in exogenous variables to obtain statistical identification (Bhargava 1991; Cameron and Trivedi 2005).

In terms of variables of main interest in the uncorrected results, the effect for the respondent’s own lagged modern contraceptive use and the effect of lagged community modern contraceptive use—one of our measures for social learning—are significant at the 0.1% level. In terms of the average marginal effects for these two lagged variables, lagged individual use has about four times the effect as lagged community use, but lagged community use has by far the second largest average marginal effect across all variables in the model. Neighboring community use has no effect, but our measure for social influence—community-level praise—has a positive and significant effect at the 10% level. As noted earlier, we are unable to backdate this variable, so we use the Year 5 value for all five years. We obtain a similar result if we allow this variable to have an effect only in Year 5.

We now turn to the joint estimation results that correct for bias. The results from Year 1 modern contraceptive use and lagged community use (Eqs. (4) and (5)) can be found in the online appendix along with the estimated heterogeneity parameters. The data support quite a bit of heterogeneity, with 6 mass points for both the community and individual-level errors (online appendix, Table A4). A chi-squared test for significant improvement in the likelihood function with the addition of the heterogeneity parameters strongly rejects the null of no improvement at the 0.1% level of significance. Therefore, there is strong evidence that joint estimation is needed in order to correct for bias.

The effects for the two key endogenous regressors—lagged individual modern contraceptive use and lagged community modern contraceptive use—have smaller marginal effects in the corrected versus uncorrected results but remain significant at the 0.1% and 5% levels, respectively. Thus, even after we control for the respondent’s own past use and the endogeneity of both individual past use and community past use, social learning as measured by lagged community-level modern contraceptive use seems to play a strong positive role in increasing modern contraceptive use.

### Simulation Results

The implication of this result for our proxy for social learning is that there appear to be social multiplier effects from the FP programs that were conducted in these two states, which gives hope that program gains can be sustained and even built upon in subsequent years. To quantify the size of these multiplier effects, we conduct a dynamic simulation that predicts the percentage of women using modern contraception each year over the five-year period. The simulation also allows us to compare the path of the modern contraceptive use increase for the model with heterogeneity corrections to uncorrected results.

The simulation proceeds as follows. We initialize modern contraceptive use to 0 for every woman in the sample in Year 1, which means that we also set community-level modern contraceptive use to 0 in Year 1. In subsequent years, we use the estimated coefficients, respondent-specific controls, and (in the case of the corrected results) the estimated heterogeneity distribution to predict the probability that woman *i* from community *j* used a modern method. We then compare this predicted probability with a random draw from a uniform distribution with endpoints 0 and 1. If, for example, the predicted probability of modern contraceptive use for an individual woman is calculated to be .05, we would assign a value of 1 to modern contraceptive use for the woman if the uniform random variable is between 0 and .05, and 0 if it is greater than .05. This process is followed for each woman in the sample. Community-level modern contraceptive use is then determined by averaging the simulated modern contraceptive use in each community where the average excludes the index woman.

Table 7 presents simulation results as predicted probabilities of modern contraceptive use. The standard errors for the predicted probabilities are calculated using a parametric bootstrap procedure described in the online appendix. For both uncorrected and heterogeneity corrected results, we use two scenarios: one with and one without social learning. The simulations without social learning simply set the coefficient for lagged community use to 0. The Year 2 simulations with and without social learning are identical because lagged community use (i.e., Year 1 community-level use) at Year 2 is 0 in all cases. With lagged community use and lagged individual modern contraceptive use set to 0 at Year 2, the predicted probability of use is determined solely by exogenous characteristics, such as age and education for the women in the sample. We see that Year 2 simulated use is much lower for the uncorrected model. This is the expected result because the estimated effects of both lagged individual use and lagged community use are overstated when we do not correct for unobserved heterogeneity or the endogeneity of these variables, so setting both variables to 0 has a more pronounced effect in the uncorrected model.

**Table 7:** Predicted probabilities (%) of modern contraceptive use based on the uncorrected and corrected estimation results from Table 6

Year	No Heterogeneity Correction		Heterogeneity Corrected	
	No Social Learning	Social Learning	No Social Learning	Social Learning
1	0.00	0.00	0.00	0.00
2	5.09	5.09	10.29	10.29
	(0.03)	(0.04)	(0.12)	(0.12)
3	7.74	8.43	13.64	14.60
	(0.18)	(0.17)	(0.23)	(0.24)
4	9.81	11.55	16.19	18.16
	(0.21)	(0.22)	(0.27)	(0.30)
5	12.06	15.46	19.22	22.14
	(0.23)	(0.27)	(0.31)	(0.34)

*Note:* Standard errors are shown in parentheses.

When we compare the no social learning to social learning columns, we see evidence of social multiplier effects for both the corrected and uncorrected results. In each subsequent year, lagged community use is larger, and it in turn stimulates higher probabilities of modern contraceptive use in subsequent years.

## Conclusion

When the Bill & Melinda Gates Foundation initially conceived of their Urban Reproductive Health Initiative, the decision was made to focus efforts to increase modern contraceptive use in major urban areas. The hope was that these cities would then be the impetus of change for surrounding areas. However, the initial evaluation design for the project did not provide a mechanism to test whether such diffusion or social multiplier effects actually occurred. This study uses endline representative data from the entire states for two program cities to study diffusion of program activities beyond the target cities. In addition, a five-year contraceptive use calendar allows us to study the effects of social learning across time, another key component of social multiplier effects, on the spread of contraceptive use.

It is well known that it is difficult to measure causal effects for demand-side programs with cross-sectional data because one typically must rely on self-reported recall of exposure. Use of self-reports can result in measures of program impact that could be biased in either direction. With cross-sectional data, the researcher typically corrects for this bias with instrumental variables methods that require the identification of variables that directly affect exposure but have only an indirect effect on contraceptive use. Finding instruments that strongly identify exposure is often difficult, and the use of weak instruments can be worse than ignoring the bias altogether (Guilkey and Lance 2014). In the absence of strong identifying variables, the approach used in this work is to compare results from three estimation strategies, two of which provide at least a partial correction for recall bias in different ways. These cross-sectional results provide evidence of diffusion of program effects beyond the target intervention sites, with the strongest evidence coming from models in which a summary measure related to attitudes toward modern contraception (praise for use) is used.

We then use the five-year calendar data to create a pseudo-longitudinal data set. With these data, we find evidence of an association between our measure of social influence and contraceptive use in a model with no correction for endogeneity bias, but we are unable to identify this effect in our corrected model. However, our measure of social learning has a strong causal effect in our dynamic model estimations. Even after we control for an individual’s lagged use of modern contraception, we find that lagged community use has a positive impact on the individual’s current use. This result is promising, indicating that momentum established during implementation of a FP program may have effects that continue beyond program termination.

Both a strength and a limitation of this work is related to the data used in the study. A major strength is that the data include not only the program cities but also the surrounding rural and other urban areas. However, better data could strengthen these analyses. First, our analyses use community averages as regressors. If the data sets included more individuals surveyed in each community, these measures would be more accurate and the influence of any one individual would be reduced. Second, because we do not have social network data, we have to use a proxy for the social network by using the community in which the respondent lives. Future studies could ask women (and men) about their social networks to obtain more refined information on social influencers. Third, we cannot get stable results using instrumental variables in the cross-sectional models. Future data collection efforts could include variables such as community-level data on transportation networks and the level of penetration of radio and TV in rural areas that could serve as potential instruments. Another remedy for measuring causal effects is the use of longitudinal data and fixed-effects methods. Longitudinal data would also obviate the need to rely on calendar data to construct the history of an individual’s contraceptive use and the creation of a pseudo-longitudinal sample as done here. Nevertheless, our statistical methods control for measurement error in both individual and community-level contraceptive use and therefore are indicative of important social learning effects that go beyond the target sites of the program.

## Electronic supplementary material

ESM 1 (DOCX 43 kb)

## Data Availability

Data from this study and all documentation are available upon request through the MLE Dataverse website at: https://dataverse.unc.edu/dataverse/mle.

## Footnotes

1 Modern contraception is defined by the following methods: female sterilization, implants, injectables, intrauterine device, daily or emergency oral contraceptive pill, male or female condom, and lactational amenorrhea.

2 As part of this earlier work, we also examined the costs of the program components and found that exposure to radio program was relatively cost-effective. More details are available from the authors upon request.

3 In the questionnaire, the praise variable was defined from the following question: Do you think there are some people within this community who will praise, encourage, or talk favorably about you if they knew that you were using a family planning/contraceptive method? (Yes, No, Don’t Know).

4 The average cluster size was 37 respondents.
