# Supplementary material for: The Examination of Diffusion Effects on Modern Contraceptive Use in Nigeria
Source: Demography. 2020 May 19;57(3):873–98. doi: 10.1007/s13524-020-00884-6 (PMC7329794; doi:10.1007/s13524-020-00884-6)
Supplement: Supplementary file 1 — (DOCX 43 kb) [file 13524_2020_884_MOESM1_ESM.docx]

**Online Appendix: Diffusion of Modern Contraception in Two Nigerian States**

**A1: Specific Survey Questions used to Define Variables Representing NURHI Program Exposure and Receiving Praise for Family Planning Use**

Participants were asked the following questions to determine exposure to NURHI components, and perception of receiving praise for modern contraceptive use:

1. Exposure to family planning messages on TV: Have you seen any family planning/child birth spacing programs/ information on the TV in the past three months?
2. Exposure to NURHI family planning messages on the radio:
3. Have you heard a radio jingle or spot with people talking about family planning or child spacing during a naming ceremony? (Yes, No, Don’t Know)
4. Have you heard a radio jingle or spot with people talking about family planning or child spacing in a hair dressing salon/ barbing salon? (Yes, No, Don’t Know)
5. Have you heard a radio jingle or spot with a family planning service provider answering questions about FP or talking to a couple? (Yes, No, Don’t Know)
6. Have you heard a radio jingle or spot with a couple talking about FP? (Yes, No, Don’t Know)
7. Have you heard a woman sharing her experience supporting the use of FP (Testimonial) on radio? (Yes, No, Don’t Know)
8. In the past year, have you heard or seen the phrase “Get it Together”? (Radio drama program/Radio jingle or spot)
9. In the past year, have you seen/heard the phrase “Know, Talk, Go”? (Radio drama program/Radio jingle or spot)
10. In the past year, have you seen/heard the phrase ““No dulling”? (Radio drama program/Radio jingle or spot)
11. In the past year, have you heard the phrase “Se o jasi” – (meaning “Are you into it?” or Are you part of it”)? (Radio drama program/Radio jingle or spot)
12. In the past year, have you heard the phrase “Mo ti feto si” – Iwo nko? (Meaning “I have planned my own, what about you?) (Radio drama program/Radio jingle or spot)
13. In the past year, have you heard the phrase “Ki la siri ewa re” – “ifeto somo bibi lasiri ewa mi” (meaning what is the secret of your beauty? Family planning is the secret of my beauty). (Radio drama program/Radio jingle or spot)
14. In the past year, have you heard the phrase “Kun gane, tazaran haihuwa” (meaning Go for family planning)? (Radio drama program/Radio jingle or spot)
15. Exposure to NURHI community outreach: In the past year, have you heard any information about family planning/child birth spacing at any of the following events: Naming Ceremonies, Freedom ceremonies from an apprenticeship, School graduation ceremonies, Christmas/Eid celebration, Wedding? (Yes, No, Don’t Know)
16. Exposure to NURHI badge: In the past year, have you seen any health provider wearing a badge/button that said “Ask me about FP?” (Yes, No, Don’t Know)
17. Receive praise for family planning use: Do you think there are some people within this community who will praise, encourage, or talk favorably about you if they knew that you were using a family planning/contraceptive method? (Yes, No, Don’t Know)

**A2: Simulation Method used for Standard Errors Reported in Table 7**

Because the simulation results are based on estimated coefficients, we added standard errors using parametric bootstrap methods. To do this, we assume that the entire set of estimated coefficients, mass points, and mass point weights follow a multivariate normal distribution centered at the estimated values of the parameters with covariance matrix equal to the estimated covariance matrix for the entire set of parameters. We then draw a set of multivariate normally distributed random variables from this distribution for the simulation exercise. We repeat this process 1,000 times. The standard deviation across the 1,000 bootstrap samples can then be used to construct standard errors reported in Table 7. The point estimates, along with the entire estimated covariance matrix, could be used to conduct formal statistical tests for differences across rows and columns of Table 7. We did not do this because the estimated standard errors were so small relative to the point estimates so every comparison would result in significant differences.

| Table A1: Cross-Sectional Model Results for Receiving Praise for Family Planning Use for Rural and Other Urban Kaduna and Oyo States: Control Variables | | | | | | | | |
| --- | --- | --- | --- | --- | --- | --- | --- | --- |
|  | Rural | | | | Other Urban | | | |
|  | Coef. | SE | Margin | SE margin | Coef. | SE | Margin | SE margin |
| Oyo state | -1.183 | 1.097 | -0.103 | 0.083 | 1.708 | 0.549*** | 0.218 | 0.068 |
| Age (years) |  |  |  |  |  |  |  |  |
| 15-19 | Ref | Ref | Ref | Ref | Ref | Ref | Ref | Ref |
| 20-24 | 0.815 | 0.316** | 0.092 | 0.039 | 1.768 | 0.317*** | 0.250 | 0.044 |
| 25-29 | 1.447 | 0.310*** | 0.179 | 0.044 | 1.994 | 0.331*** | 0.290 | 0.047 |
| 30-34 | 1.306 | 0.320*** | 0.161 | 0.045 | 2.055 | 0.338*** | 0.304 | 0.049 |
| 35-39 | 1.511 | 0.323*** | 0.195 | 0.049 | 2.154 | 0.345*** | 0.327 | 0.052 |
| 40-44 | 1.360 | 0.342*** | 0.175 | 0.052 | 2.158 | 0.347*** | 0.332 | 0.053 |
| 45-49 | 0.926 | 0.374* | 0.112 | 0.052 | 1.835 | 0.366*** | 0.279 | 0.058 |
| Education |  |  |  |  |  |  |  |  |
| None | Ref | Ref | Ref | Ref | Ref | Ref | Ref | Ref |
| Primary | 0.557 | 0.202** | 0.060 | 0.023 | 0.640 | 0.235** | 0.087 | 0.034 |
| Junior secondary | 0.561 | 0.252* | 0.063 | 0.031 | 0.506 | 0.288† | 0.069 | 0.042 |
| Senior secondary | 0.511 | 0.248* | 0.056 | 0.029 | 0.583 | 0.245* | 0.077 | 0.034 |
| Higher | 1.238 | 0.477** | 0.162 | 0.076 | 0.779 | 0.269** | 0.108 | 0.040 |
| In Union | 1.097 | 0.275*** | 0.089 | 0.017 | 0.534 | 0.189** | 0.064 | 0.021 |
| Muslim | -1.109 | 0.205*** | -0.109 | 0.020 | -0.299 | 0.145* | -0.039 | 0.019 |
| Primary language spoken at home |  |  |  |  |  |  |  |  |
| Hausa | Ref | Ref | Ref | Ref | Ref | Ref | Ref | Ref |
| Yoruba | 0.941 | 1.092 | 0.106 | 0.136 | -0.818 | 0.550 | -0.100 | 0.064 |
| English | 0.595 | 0.566 | 0.069 | 0.074 | -1.068 | 0.482* | -0.111 | 0.039 |
| Wealth |  |  |  |  |  |  |  |  |
| Poorest | 1.322 | 0.736† | 0.143 | 0.083 | -0.619 | 0.384 | -0.071 | 0.039 |
| Poor | 1.554 | 0.732* | 0.178 | 0.093 | -0.639 | 0.299* | -0.074 | 0.031 |
| Middle | 1.883 | 0.726** | 0.240 | 0.108 | 0.021 | 0.200 | 0.003 | 0.026 |
| Rich | 1.639 | 0.729* | 0.225 | 0.122 | 0.226 | 0.166 | 0.029 | 0.022 |
| Richest | Ref | Ref | Ref | Ref | Ref | Ref | Ref | Ref |
| $\sigma_{\mu}$ | 0.506 | 0.410 |  |  | 0.495 | 0.103 |  |  |
| Sample size | 2,330 |  |  |  | 2,245 |  |  |  |

†p < .10; *p < .05; **p < .01; ***p<0.001

Note: Coefficients presented are from individual exposure model but are representative of all 3 models.

Standard errors for marginal effects presented in parentheses.

| Table A2: Cross-Sectional Model Results for Rural and Other Urban Kaduna and Oyo States: Receiving Praise for Family Planning Use as a Function of NURHI Components | | | | | | | | | | | | | | |
| --- | --- | --- | --- | --- | --- | --- | --- | --- | --- | --- | --- | --- | --- | --- |
|  | Model (a)  Individual exposure | | | | | Model (b)  Community Exposure | | | | Model (c)  Correlated Random Effects | | | | |
|  | Coef. | SE | Margin | | SE margin | Coef. | SE | Margin | SE margin | Coef. | SE | Margin | SE margin |  |
| Rural model exposures | | | | | | | | | | | | | | |
| Radio | 0.937*** | 0.112 | 0.196 | 0.024 | | 1.656*** | 0.456 | 0.349 | 0.095 | 0.885*** | 0.115 | 0.183 | 0.024 |  |
| Outreach | 0.626*** | 0.117 | 0.129 | 0.024 | | -0.927* | 0.548 | -0.195 | 0.115 | 0.684*** | 0.120 | 0.139 | 0.025 |  |
| Badge | 0.832* | 0.309 | 0.168 | 0.061 | | -0.760 | 1.443 | -0.160 | 0.304 | 0.817** | 0.313 | 0.163 | 0.061 |  |
| TV | 0.252 | 0.286 | 0.051 | 0.058 | | 0.900 | 1.361 | 0.190 | 0.286 | 0.227 | 0.291 | 0.045 | 0.058 |  |
| Traveled to  NURHI program city | 0.174 | 0.185 | 0.035 | 0.037 | | -0.213 | 0.940 | -0.045 | 0.198 | 0.222 | 0.189 | 0.044 | 0.038 |  |
| Other Urban model exposures | | | | | | | | | | | | | |  |
| Radio | 0.177 | 0.157 | 0.022 | 0.020 | | 0.314 | 0.689 | 0.068 | 0.149 | 0.458*** | 0.115 | 0.098 | 0.025 |  |
| Outreach | -0.185 | 0.143 | -0.023 | 0.018 | | -1.941 | 0.765* | -0.420 | 0.160 | 1.006*** | 0.116 | 0.214 | 0.023 |  |
| Badge | 0.590*** | 0.164 | 0.082 | 0.024 | | 0.049 | 0.777 | 0.011 | 0.168 | 0.091 | 0.159 | 0.019 | 0.033 |  |
| TV | -0.113 | 0.142 | -0.014 | 0.018 | | -0.238 | 0.762 | -0.051 | 0.165 | 0.143 | 0.129 | 0.030 | 0.027 |  |
| Traveled to NURHI  program city | -0.001 | 0.189 | 0.000 | 0.024 | | 0.043 | 1.038 | 0.009 | 0.225 | 0.020 | 0.150 | 0.004 | 0.031 |  |

†p < .10; *p < .05; **p < .01; ***p<0.001

Notes: Model (b) estimates for mean community level variables and average marginal effects. Model (c) presents within estimates.

| Table A3: Pseudo Longitudinal Multivariate Results for the Pooled Data: Auxiliary Results | | | | | | | | |
| --- | --- | --- | --- | --- | --- | --- | --- | --- |
|  | Time 1 mCPR | | | | Community mCPR | | | |
|  | Coef. | SE | Z | P-value | Coef. | SE | Z | P-value |
| Constant | -12.6252 | 0.6533 | -19.32 | <0.01 | 0.6111 | 0.0557 | 10.98 | <0.01 |
| Age (years) | 0.5925 | 0.0610 | 9.71 | <0.01 | -0.0265 | 0.0047 | -5.63 | <0.01 |
| Age Squared | -0.0091 | 0.0010 | -8.91 | <0.01 | 0.0004 | 0.0001 | 4.63 | <0.01 |
| Education |  |  |  |  |  |  |  |  |
| None | Ref | Ref | Ref | Ref | Ref | Ref | Ref | Ref |
| Primary | 0.5686 | 0.2238 | 3.90 | <0.01 | 0.0028 | 0.0107 | 0.26 | 0.79 |
| Junior secondary | 0.8727 | 0.2238 | 3.90 | <0.01 | 0.1057 | 0.0161 | 6.57 | <0.01 |
| Senior secondary | 0.9531 | 0.2100 | 4.54 | <0.01 | 0.2136 | 0.0157 | 13.44 | <0.01 |
| Higher | 1.1231 | 0.2311 | 4.86 | <0.01 | 0.0620 | 0.0154 | 4.03 | <0.01 |
| In Union | 1.2616 | 0.1783 | 7.08 | <0.01 | 0.0100 | 0.0189 | 0.54 | 0.59 |
| Muslim | -0.5672 | 0.1310 | -4.33 | <0.01 | -0.0530 | 0.0050 | -10.67 | <0.01 |
| Kaduna other urban | Ref | Ref | Ref | Ref | Ref | Ref | Ref | Ref |
| Oyo rural | 0.3137 | 0.2910 | 1.08 | 0.28 | -0.0259 | 0.0048 | -5.44 | <0.01 |
| Kaduna Rural | -0.2606 | 0.2563 | -1.02 | 0.31 | -0.0761 | 0.0045 | -16.88 | <0.01 |
| Oyo other urban | 2.0145 | 0.2658 | 7.58 | <0.01 | 0.1422 | 0.0041 | 34.91 | <0.01 |
| Kaduna | 1.2947 | 0.2470 | 5.24 | <0.01 | 0.0470 | 0.0039 | 12.18 | <0.01 |
| Ibadan | 2.3490 | 0.2818 | 8.34 | <0.01 | 0.1805 | 0.0052 | 34.64 | <0.01 |
| Always a resident | 0.3900 | 0.1135 | 3.44 | <0.01 | 0.0939 | 0.0069 | 13.57 | <0.01 |
| Year 2 | Ref | Ref | Ref | Ref | Ref | Ref | Ref | Ref |
| Year 3 |  |  |  |  | 0.0380 | 0.0032 | 11.93 | <0.01 |
| Year 4 |  |  |  |  | 0.0599 | 0.0044 | 13.54 | <0.01 |
| Year 5 |  |  |  |  | 0.0849 | 0.0052 | 16.18 | <0.01 |

| Table A4: Heterogeneity Parameters | | | | | | | |
| --- | --- | --- | --- | --- | --- | --- | --- |
|  | Coef. | SE | Z | P-value |  | Probability Weights | |
| Modern mCPR | | | | |  |  |  |
| Community 1 | 0 |  |  |  |  | Community 1 | 0.1179 |
| Community 2 | 0.2959 | 0.1079 | 2.74 | <0.01 |  | Community 2 | 0.1397 |
| Community 3 | -0.5910 | 0.1292 | -4.57 | <0.01 |  | Community 3 | 0.1485 |
| Community 4 | -0.3544 | 0.0991 | 3.58 | <0.01 |  | Community 4 | 0.1790 |
| Community 5 | -0.8865 | 0.1342 | -6.61 | <0.01 |  | Community 5 | 0.2096 |
| Community 6 | -1.2832 | 0.1596 | -8.04 | <0.01 |  | Community 6 | 0.2053 |
| Individual 1 | 0 |  |  |  |  | Individual 1 | 0.0048 |
| Individual 2 | -1.5123 | 0.4507 | -3.36 | <0.01 |  | Individual 2 | 0.4157 |
| Individual 3 | 0.9684 | 0.4175 | 2.32 | 0.02 |  | Individual 3 | 0.4739 |
| Individual 4 | 0.4593 | 0.5614 | 0.82 | 0.41 |  | Individual 4 | 0.0153 |
| Individual 5 | 27.5551 | NA | NA | NA |  | Individual 5 | 0.0001 |
| Individual 6 | 3.0518 | 0.4666 | 6.54 | <0.01 |  | Individual 6 | 0.0903 |
| Time Period 1 mCPR | | | | |  |  |  |
| Community 1 | 0 |  |  |  |  |  |  |
| Community 2 | 0.7497 | 0.1796 | 4.18 | <0.01 |  |  |  |
| Community 3 | -0.7449 | 0.2033 | -3.66 | <0.01 |  |  |  |
| Community 4 | -0.5944 | 0.1711 | -3.74 | <0.01 |  |  |  |
| Community 5 | -1.2604 | 0.2012 | -6.27 | <0.01 |  |  |  |
| Community 6 | -2.2662 | 0.2407 | -9.42 | <0.01 |  |  |  |
| Individual 1 | 0 |  |  |  |  |  |  |
| Individual 2 | -2.7337 | 0.4091 | -6.68 | <0.01 |  |  |  |
| Individual 3 | 0.3145 | 0.3711 | 0.85 | 0.40 |  |  |  |
| Individual 4 | 0.9572 | 0.6306 | 1.52 | 0.13 |  |  |  |
| Individual 5 | 26.6602 | NA | NA | NA |  |  |  |
| Individual 6 | 3.6758 | 0.3714 | 9.90 | <0.01 |  |  |  |
| Community mCPR | | | | |  |  |  |
| Community 1 | 0 |  |  |  |  |  |  |
| Community 2 | 0.0843 | 0.0070 | 12.05 | <0.01 |  |  |  |
| Community 3 | -0.0910 | 0.0034 | -26.58 | <0.01 |  |  |  |
| Community 4 | -0.0544 | 0.0035 | -15.41 | <0.01 |  |  |  |
| Community 5 | -0.1324 | 0.0037 | -36.28 | <0.01 |  |  |  |
| Community 6 | -0.1934 | 0.0046 | -41.83 | <0.01 |  |  |  |
| Individual 1 | 0 |  |  |  |  |  |  |
| Individual 2 | -0.0927 | 0.0100 | -9.29 | <0.01 |  |  |  |
| Individual 3 | -0.1014 | 0.0101 | -10.01 | <0.01 |  |  |  |
| Individual 4 | -0.1819 | 0.0122 | 14.87 | <0.01 |  |  |  |
| Individual 5 | -0.2391 | 0.0126 | -19.01 | <0.01 |  |  |  |
| Individual 6 | -0.1188 | 0.0105 | -11.35 | <0.01 |  |  |  |
